# Drainage-system development in consecutive melt seasons at a polythermal, Arctic glacier, evaluated by flow-recession analysis and linear-reservoir simulation

**DOI:** 10.1002/wrcr.20257

**Published:** 2013-07-26

**Authors:** Richard Hodgkins, Richard Cooper, Martyn Tranter, Jemma Wadham

**Affiliations:** 1 Department of Geography, Loughborough UniversityLoughborough, Leicestershire, UK; 2 School of Geographical Sciences, University of BristolBristol, UK

## Abstract

[1] The drainage systems of polythermal glaciers play an important role in high-latitude hydrology, and are determinants of ice flow rate. Flow-recession analysis and linear-reservoir simulation of runoff time series are here used to evaluate seasonal and inter-annual variability in the drainage system of the polythermal Finsterwalderbreen, Svalbard, in 1999 and 2000. Linear-flow recessions are pervasive, with mean coefficients of a fast reservoir varying from 16 (1999) to 41 h (2000), and mean coefficients of an intermittent, slow reservoir varying from 54 (1999) to 114 h (2000). Drainage-system efficiency is greater overall in the first of the two seasons, the simplest explanation of which is more rapid depletion of the snow cover. Reservoir coefficients generally decline during each season (at 0.22 h d^−1^ in 1999 and 0.52 h d^−1^ in 2000), denoting an increase in drainage efficiency. However, coefficients do not exhibit a consistent relationship with discharge. Finsterwalderbreen therefore appears to behave as an intermediate case between temperate glaciers and other polythermal glaciers with smaller proportions of temperate ice. Linear-reservoir runoff simulations exhibit limited sensitivity to a relatively wide range of reservoir coefficients, although the use of fixed coefficients in a spatially lumped model can generate significant subseasonal error. At Finsterwalderbreen, an ice-marginal channel with the characteristics of a fast reservoir, and a subglacial upwelling with the characteristics of a slow reservoir, both route meltwater to the terminus. This suggests that drainage-system components of significantly contrasting efficiencies can coexist spatially and temporally at polythermal glaciers.

## 1. Introduction

[2] Glaciers play a critical role in the water cycle of high latitudes and high altitudes, heavily modulating the catchment-scale relationship between precipitation and runoff [*Röthlisberger and Lang*, 1987]. Glacier drainage systems are also a major driver of ice dynamics at scales ranging from individual mountain glaciers [*Anderson et al*., 2004] to ice sheets [*Bartholomew et al*., 2010]. Nevertheless, investigations of glacier drainage systems remain challenged by issues of remoteness and intractability, even before the fundamental inaccessibility of water flow beneath the ice surface is considered. Yet there is a need to improve understanding of polythermal glacier hydrology in particular, since nontemperate ice (ice below the pressure-melting temperature, lacking interstitial water) is commonly encountered in high-latitude ice masses [*Irvine-Fynn et al*., 2011]. In principle an aquiclude, nontemperate ice can be distributed through high-latitude glaciers in different proportions and locations [*Blatter and Hutter*, 1991; *Irvine-Fynn et al*., 2011], adding potential complexity to the routing of meltwater compared with wholly temperate glaciers. Furthermore, it is conceivable that atmospheric warming could either decrease or increase the proportion of nontemperate ice in high-latitude glaciers, depending on the specific interaction of ice geometry and local climate [*Irvine-Fynn et al*., 2011].

[3] Given the challenges of instrumenting glaciers, insights into their drainage have often been sought from analyses of their hydrological outputs, such as the dissolved constituents of meltwater [e.g., *Wadham et al*., 2000] and proglacial hydrograph forms [e.g.. *Hannah et al*., 1999]. The foundation of these approaches is the notion that the composition or form of the proglacial meltwater flow reflects the characteristics of the glacier's drainage system, and therefore that the proglacial hydrograph can be a valuable source of information on the general routing of meltwater. Models of glacier hydrology have been used to estimate water resources [e.g., *Escher-Vetter*, 2000], to quantify geomorphological or biogeochemical processes [e.g., *Richards et al*., 1996], to assess hydroecological status [e.g., *Brown et al*., 2010], and to investigate drainage-system structure, its seasonal change, and the influence of that change on water storage and runoff patterns [e.g., *Flowers*, 2008].

[4] Glaciers evolve different drainage structures to accommodate water flows of different magnitudes, with most systems featuring a fast-draining, high-flow component and/or a slow-draining, low-flow component [*Fountain and Walder*, 1998]. Such components can be conceptualized in various combinations, such as episodic icemelt and diffuse snowmelt when considering the glacier generally, or channels and linked cavities when considering the subglacial environment in particular. This conceptualization should be equally applicable to both temperate and polythermal glaciers, since features such as snow or firn aquifers, permeable subglacial sediments, or even a near-surface percolation layer [*Irvine-Fynn et al*., 2011], would yield a slow-drainage component to complement the fast, channelized subaerial or subglacial flow of even the simplest drainage systems.

[5] The overall aim of this paper is therefore to investigate the drainage system of a polythermal glacier, by quantifying the seasonal and inter-annual variability of meltwater throughflow rates determined from the proglacial hydrograph. The approach taken is to use flow-recession analysis [*Sujono et al*., 2004] and linear-reservoir modeling [*Chow et al*., 1998]; reviews of the application of linear-reservoir modeling to glacier hydrology have been provided by *Jansson et al*. [2003] and *Hock and Jansson* [2005]. Specifically, the methodology is: (1) a flow-recession analysis of two, consecutive melt-seasons' runoff data from the glacier Finsterwalderbreen, Svalbard; (2) linear-reservoir modeling of runoff from the glacier, in order to acquire insight into its drainage system, and to draw inferences about the wider applicability of this approach to polythermal glaciers in general; (3) a synthesis of the results from (1) and (2) in the context of temporal variability and glacier thermal regime, with a view to drawing inferences about the structure of the drainage system.

## 2. Data and Methods

### 2.1 Data Collection Methods

**Figure 1:**
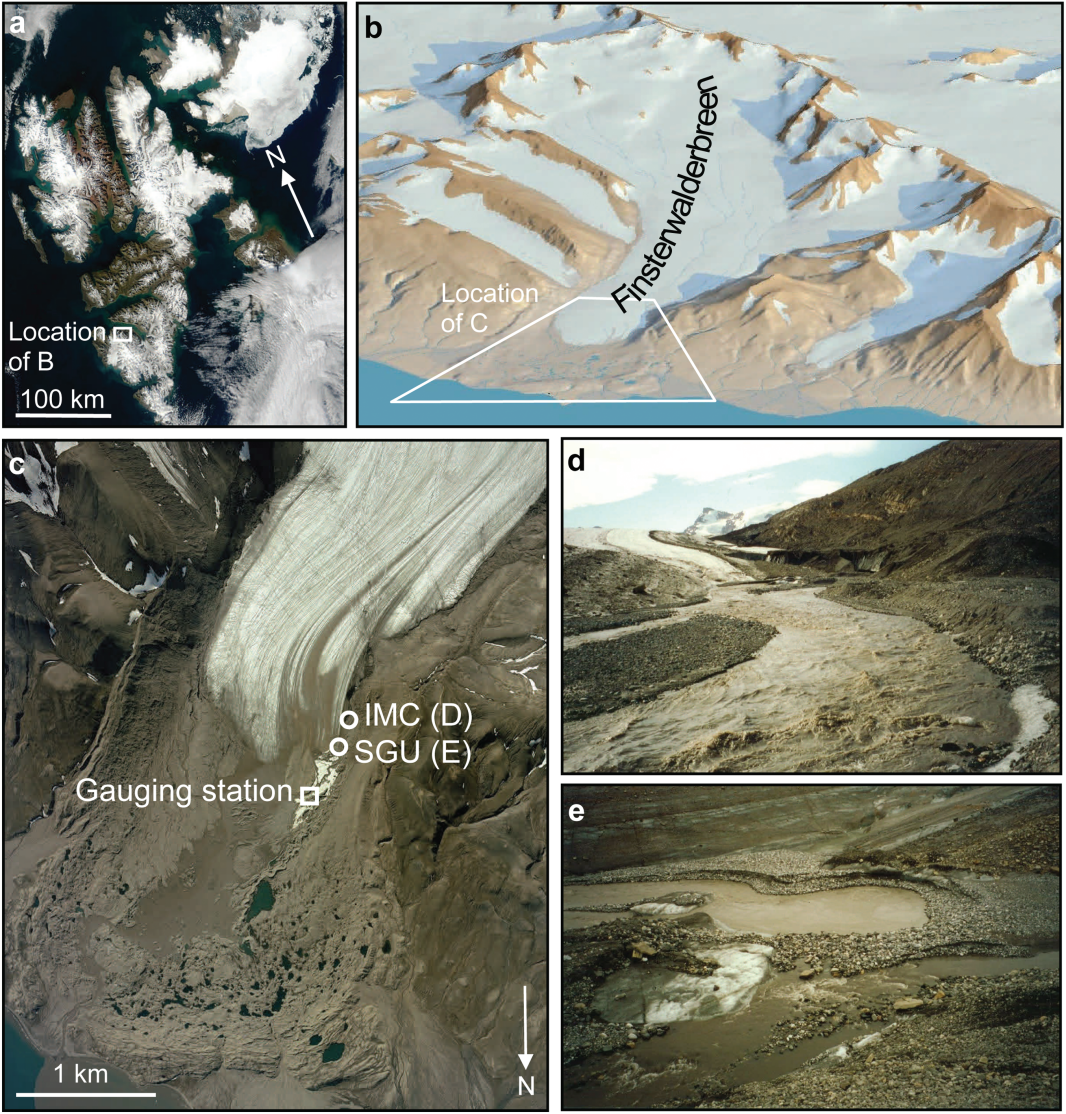
(a) Location of Finsterwalderbreen within the Svalbard archipelago; (b) perspective view of the Finsterwalderbreen catchment from the north (©Norwegian Polar Institute, TopoSvalbard); (c) aerial view of Finsterwalderbeen terminus and proglacial area(©UK Natural Environment Research Council, Airborne Research and Survey Facility, 2003), showing locations of the runoff gauging station and of the ice-marginal channel outfall (IMC) and subglacial upwelling (SGU); (d) the ice-marginal channel outfall at the western margin of the glacier; (e) the subglacial upwelling 2013.

[6] The studied glacier, Finsterwalderbreen, is located at 77° 31′ N, 15° 19′ E in southern Spitsbergen, the largest island of the Norwegian Arctic archipelago of Svalbard (Figure 1). The glacier itself is 12 km long, north facing, and flows to the coast from a maximum elevation of 1065 m a.s.l. The glacier is up to 200 m thick, and has a polythermal temperature structure, with a cold surface layer 25–170 m thick, a warm firn accumulation zone and a bed which is mostly temperate, apart from limited areas at the margins [*Ødegård et al*., 1997]. Since its most recent maximum extent, between 1898 and 1918, the glacier terminus has thinned and retreated at a rate of 10–45 m a^−1^ [*Nuttall et al*., 1997]. The geometry, flow, mass balance and hydrology of Finsterwalderbreen are reasonably well documented [e.g., *Cooper et al*., 2011; *Hodgkins et al*. 2005,^2007^; *Nuttall and Hodgkins*, 2005; *Pinglot et al*., 1997; *Wadham et al*., 2001].

[7] Meltwater from the glacier issues from both margins at the terminus, but the majority is routed to the west as a result of the glacier's surface profile: *Hagen et al*. [2000] estimated the area draining to the west at 32 km^2^ (3 km^2^ to the east) from a 1990 DEM. Evidence suggests that meltwater flows subglacially at Finsterwalderbreen: *Wadham et al*. [2010] suggested that two systems contribute meltwater to the main runoff at the western margin: a long-residence time (several days) system feeding an artesian subglacial upwelling outflow, and a shorter-residence time (several hours) channelized-drainage system, culminating in a subaerial, ice-marginal channel (Figure 1). Nontemperate ice at the glacier front probably forces some meltwater into a talik-like, underground flow, which subsequently emerges near the terminus as the upwelling feature (Figure 1). Similar bipartite structures have been inferred at other polythermal glaciers [e.g., *Irvine-Fynn et al*., 2005; *Pälli et al*., 2003; *Skidmore and Sharp*, 1999; *Vatne et al*., 1996], underlining the distinctive hydrology of such glaciers.

[8] This study is based on discharge time series from the west drainage system obtained in 1999 and 2000, described in detail in *Hodgkins et al*. [2009]. Discharge was monitored in the same, quasi-stable reach over the intervals 17:00 24 June–09:00 17 August 1999, and 12:00 27 June–12:00 12 August 2000. Meltwater from the upwelling mixes with the ice-marginal runoff upstream of the monitoring point (Figure 1). The probable error in discharge was estimated as a function of potential errors in the continuous measurement of stage, in discrete measurements of flow velocity and channel depth, and in the rating curves used to convert stage to discharge, at ±14.3–18.4% in 1999 and ±11.4–23.7% in 2000 [*Cooper*, 2003; *Hodgkins et al*., 2009]. The range of values is mainly a consequence of the need to change rating curves as reach geometry altered. There was no discernible difference in the configuration of the west drainage outfall between the 2 years of monitoring.

### 2.2 Data Analysis Methods

[9] For the reasons stated in the introduction, linear-reservoir models often assume two principal hydrological pathways or reservoirs: a fast one (which accommodates high flows) and a slow one (which accommodates low flows). The former would typically represent icemelt drained through an efficient, channelized system; the latter would typically represent snowmelt drained through an inefficient, distributed system [*Fountain and Walder*, 1998]. An important characteristic of this approach is that the drainage system is broken down in a conceptual way, without explicit representation of specific, physical components or process interactions. While seemingly a coarse approach to differentiating drainage, this implicitly links process, state and flux to retain the most important characteristics of the major drainage pathways: for instance, for a fast-melting, fast-flow pathway with high-magnitude outflow, the cascade from melt to runoff is entirely integrated.

[10] The linear-reservoir approach is based on relating stored water volume, *V*, to the rate of outflow (discharge or runoff), *Q* [*Chow et al*., 1998]: 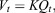
(1)where *t* denotes the time step and *K* is commonly referred to as a *storage constant*, although the term *reservoir coefficient* is preferred here, as it provides a clearer description of the role of *K* in the model. The continuity equation is then simply: 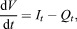
(2)indicating that the rate of change of water storage is equal to the difference between the rates of inflow, *I*, and outflow: water is effectively stored whenever the former exceeds the latter, which can occur on a wide range of spatial and temporal scales. Combining equations (1 and 2) gives: 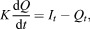
(3)which rewrites storage change in terms of outflow and the reservoir coefficient, and when integrated gives expressions for *recession flow* and *recharge flow*, explained below.

[11] It is necessary to specify a reservoir coefficient for each reservoir: this essentially describes how much of a delay each reservoir imposes on the inflow. The combined effect of the number of reservoirs and their coefficients defines the temporal pattern of outflow, expressed in the form of the hydrograph. A range of reservoir coefficient values has been published [*Hock and Jansson*, 2005], but there is considerable variation from glacier to glacier. There are very few published coefficients from studies in Svalbard [*Rutter et al*., 2011], and only a few from polythermal glaciers in other, high-latitude locations [*Hock and Noetzli*, 1997]. Reservoir coefficients are either obtained by tuning, that is, maximizing the agreement between modeled and measured glacier outflow [e.g., *Hock and Noetzli*, 1997; *Klok et al*., 2001], or by flow-recession analysis [e.g., *Gurnell*, 1993; *Hannah and Gurnell*, 2001]. Both approaches have merits and limitations, but recession analysis [*Sujono et al*., 2004] has the important benefit of deriving an estimate of reservoir coefficients independent of the modeling procedure.

[12] *Reservoir coefficients* (*K*, with units of hours) can be estimated from: 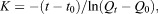
(4)where *t*_0_ is the time step preceding time *t*. This requires a knowledge of the hydrograph, so that the timing of, and discharge at, the onset and cessation of the flow recession can be defined. During periods when there is no recharge (fresh inflow) to the reservoir, the outflow at any time step (*Q_t_*) can be expressed as a function of the preceding flow (*Q*_0_) and the reservoir coefficient: 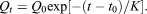
(5)

[13] This implies that during periods of recession flow, the value of *K* can be estimated from the slope of a semilogarithmic plot of discharge against time, where recessions generated by outflow from different reservoirs will plot as straight lines; identification of more than one linear component, separated by a break of slope, is generally interpreted to represent recessions from different reservoirs with different coefficients [*Gurnell*, 1993].

[14] Equation(5) defines the *recession flow*. If all melting (and other inputs such as rainfall) ceased, this would describe the runoff from the glacier. Actual runoff will consist of this recession flow, plus a *recharge flow* from ongoing inputs, defined as: 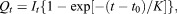
(6)which has the same exponent as the reservoir flow, but depends on inflow at the current time step, whereas reservoir flow depends on outflow at the previous time step. Combining equations (5) and (6) defines the simple, linear-reservoir model of drainage: 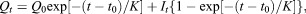
(7)which is the reservoir flow plus the recharge flow for a single reservoir; at least one more reservoir would often be employed for a complete glacier model, for the reasons stated at the start of this section and in the introduction. Typically, the reservoirs are conceptualized in parallel, meaning they both contribute directly to runoff. Such an arrangement would appear to be applicable to Finsterwalderbreen, where two meltwater systems emerge at the terminus (Figure 1).

[15] Equation (7) is used as the basis for simulations of runoff from Finsterwalderbreen. Simulation performance is assessed in three ways. *Mean Error* (*ME*) reflects the overall tendency of modeled runoff, Q*, to underestimate (if ME is positive) or overestimate (if ME is negative) measured runoff, *Q*: 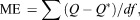
(8)where *df* is degrees of freedom, determined as *N* – *P* – 1, where *N* is the number in the sample and *P* is the number of predictors. *Root Mean Square Error* (*RMSE*) provides the standardized, mean model error for runoff: 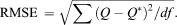
(9)

[16] The *Nash-Sutcliffe efficiency criterion*, *E*, provides an assessment of the goodness-of-fit of the modeled time series to the observed one: 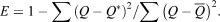
(10)

[17] The range of *E* lies between 1.0 (perfect fit) and −∞. An efficiency of lower than zero indicates that the mean value of the observed time series would have been a better predictor than the model [*Krause et al*., 2005].

## 3. Results

### 3.1 Flow-Recession Analysis

**Figure 2:**
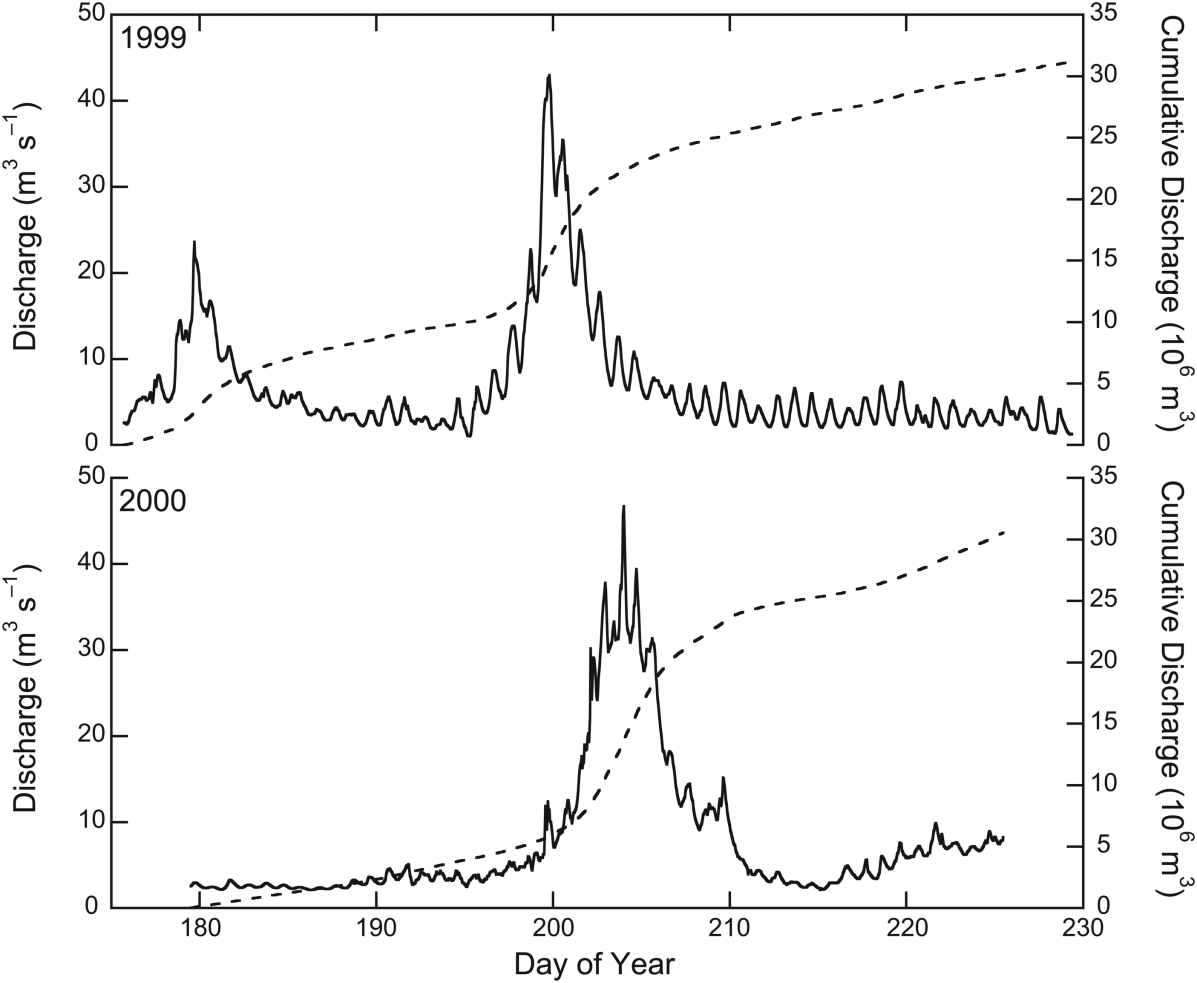
Discharge (solid line) and cumulative discharge (dashed line) time series measured at the western margin of the glacier, 1999 and 2000. Meltwater flow in 1999 varies from 1.0 to 43 m^3^ s^−1^, with a mean of 6.7±6.7 m^3^ s^−1^ (where the uncertainty term is 1*σ*); for 2000, the corresponding values are 2.1–47 m^3^ s^−1^ and 7.7±8.3 m^3^ s^−1^.

[18] The discharge time series are presented in Figure 2. In 1999, a total of 31×10^6^ m^3^ of meltwater was discharged in 1289 h, yielding a mean daily flow of 0.58×10^6^ m^3^ d^−1^. In 2000, a similar total was discharged in 1105 h, giving a mean daily flow of 0.66×10^6^ m^3^ d^−1^. The totals measured here fall within the range measured in the same location in 1994 (56 d) and 1995 (51 d) by *Hodson et al*. [1997], of 24−57×10^6^ m^3^.

**Figure 3:**
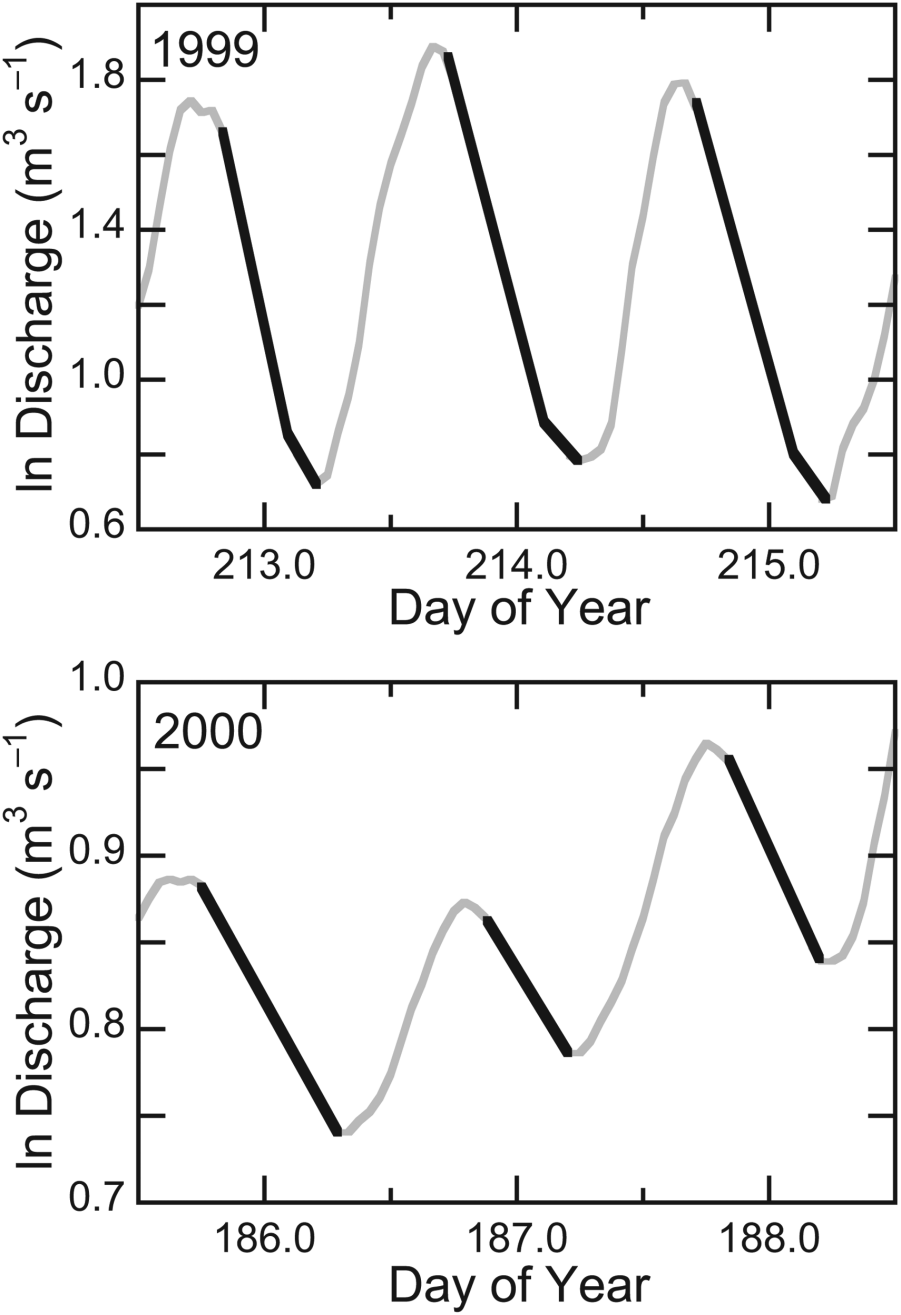
Sample flow recessions, 1999 (two-reservoir recessions) and 2000 (one-reservoir recessions). Linear-regression slope/intercept/*R*^2^ for the three recessions in 1999, where Day of Year is the independent variable and ln*Q* is the dependent variable, are: (day 212) −2.98/+635/0.99 and −0.98/+210/0.99; (day 213) −2.58/+553/1.00 and −0.61/+132/0.95; (day 214) −2.39/+514/1.00 and −0.98/+210/0.99. Corresponding values for 2000: (day 185) −0.28/+52/0.99; (day 186) −0.23/+44/0.99; (day 187) −0.33/+62/0.98. Note the consistency of the regression coefficients and the strength of the linear fit.

[19] Every flow recession of 4 h or greater in both time series was examined to determine whether it exhibited one or more linear components, which could be interpreted as drainage from specific reservoirs. Shorter periods of flow decrease were considered too brief to draw valid inferences: given the hourly resolution of the series, their analysis would have required estimating regressions from only 2–3 data points. No distinction was made between days with or without rainfall, as rainfall makes only a minor contribution to water inputs during the melt season in this location [*Cooper et al*., 2011]. Sample flow recessions are shown in Figure 3, with a run of 3 days, each showing a two-reservoir recession, from 1999, and a run of 3 days, each showing a one-reservoir recession, from 2000. Linear, ordinary least-squares regression lines have been fitted to each of the linear sections in both cases. The *R*^2^ values of the fits for the first (fast) reservoirs in 1999 vary from 0.99 to 1.0 while the fits for the second (slow) reservoirs vary from 0.95 to 0.99, indicating that the linear approach is at least a very good approximation. For the single reservoir recessions in the 2000 example, the *R*^2^ values of the fits vary from 0.98 to 0.99.

[20] Maximum recession durations (both reservoirs combined) are 17 h (1999) and 20 h (2000); maximum flow decreases are 14 m^3^ s^−1^ (1999) and 16 m^3^ s^−1^ (2000). The overall results of the flow-recession analysis, broken down by reservoir, are summarized in Table 1. Recession duration and flow decrease magnitude are positively correlated (*R* = 0.55, *p* < 0.05) in 1999, and negatively correlated (*R* = −0.35, *p* < 0.05) in 2000, for low values of flow decrease (< 5 m^3^ s^−1^); the relationships break down at greater magnitudes of flow decrease, which are not associated with the longest recessions.

**Table 1:** Summary of Flow-Recession Statistics From the 1999 and 2000 Discharge Time Series (Figure 2)^a^

Flow Recession Statistic	1999 (54 days series)	2000 (46 days series)
Reservoir 1		
*n*	50	38
Duration mean (h)	9	9
Duration maximum (h)	16	20
Δ*Q* mean (m^3^ s^−1^)	−3.5	−2.3
Δ*Q* range (m^3^ s^−1^)	−0.76 to −13	−0.20 to −13
*K*_1_ mean (h)	16	41
*K*_1_ range (h)	6–31	8–114
Flow proportion mean (%)	0.95	0.98
Reservoir 2		
*n*	31	7
Duration mean (h)	3	4
Duration maximum (h)	6	5
Δ*Q* mean (m^3^ s^−1^)	−0.35	−0.75
Δ*Q* range (m^3^ s^−1^)	−0.027 to −1.3	−0.04 to −2.8
*K*_2_ mean (h)	54	114
*K*_2_ range (h)	17–154	58–170
Flow proportion mean (%)	0.05	0.02

a *ΔQ* is the change in reservoir discharge.

[21] In 1999, 50 days from the total of 54 showed flow recessions with at least one linear component, and 31 showed recessions with two such components (Table 1). For 2000, 38 days from the total of 46 showed flow recessions with at least one linear component, but only 7 showed recessions with two such components (Table 1). There is no evidence for more than two components in any recession. Two-reservoir recessions are therefore the norm in 1999, but one-reservoir recessions are typical of 2000. The two-reservoir recessions consist of a fast component followed by a slow component, whereas the one-reservoir recessions are composed of the fast component only.

[22] *Gurnell* [1993] observed that flow recessions at Haut Glacier d'Arolla, Switzerland, typically exhibited a break of slope, separating fast and slow components of the recession, and that when a break of slope was absent, it was usually early in the ablation season and the recession present appeared to be a slow-component one. In contrast, there are no instances in the Finsterwalderbreen series where the slow reservoir is present, without the fast one. However, monitoring in both 1999 and 2000 started some time after significant depletion of the snow cover on the lower glacier and the onset of runoff, so the existence of such a pattern cannot be excluded here. On the other hand, there are numerous recessions where a fast component but no slow component is identified, particularly in 2000. Again, this does not preclude the presence of the slow component at these times: it may instead be that the fast-component recession is not complete before the next hydrograph rise.

### 3.2 Reservoir Coefficients

[23] Table1 also shows the values of the reservoir coefficients, *K*_1_ representing the fast reservoir and *K*_2_ the slow reservoir. These are determined with equation(4), from the duration and magnitude of the appropriate flow recessions. It is apparent that the values of each coefficient change throughout the respective melt seasons, and that the coefficients change appreciably from season to season. In 1999, the mean value of *K*_1_ was 16±6 h, while the mean value of *K*_2_ was 54±33 h. By contrast, in 2000 the mean value of *K*_1_ was somewhat greater at 41±23 h, as was the mean value of *K*_2_, at 114±45 h. Therefore, the 1999 melt season was characterized not only by two-reservoir recessions, but also by faster reservoir coefficients, while 2000 exhibited mainly single-reservoir recessions with slower coefficients. For comparison, *Gurnell* [1993] and *Richards et al*. [1996] identified up to four linear reservoirs at the temperate Haut Glacier d'Arolla, Switzerland, with *K* = 12–13 h, 27–29 h, 72 h and 203 h; from Svalbard, *Rutter et al*. [2011] found that two linear reservoirs were apparent for about half of the melt season at the nontemperate Rieperbreen-Foxfonna, with *K* = 63 h and 331 h.

[24] In both years, the proportion of total flow from reservoir 1 (the fast reservoir) is far greater than that from reservoir 2 (the slow reservoir). The proportional contribution of the flow decrease in each reservoir to the total flow decrease during each recession (Table1) allows the proportion of total flow in the reservoirs to be approximated. For 1999, the proportion of flow in reservoir 1 estimated in this way reached a minimum of 0.73 but had a mean value of 0.95; for 2000, the corresponding values were 0.74 and 0.98. So, even when two-reservoir recessions were frequent in 1999, the contribution of reservoir 2 to the total outflow was still very small.

**Figure 4:**
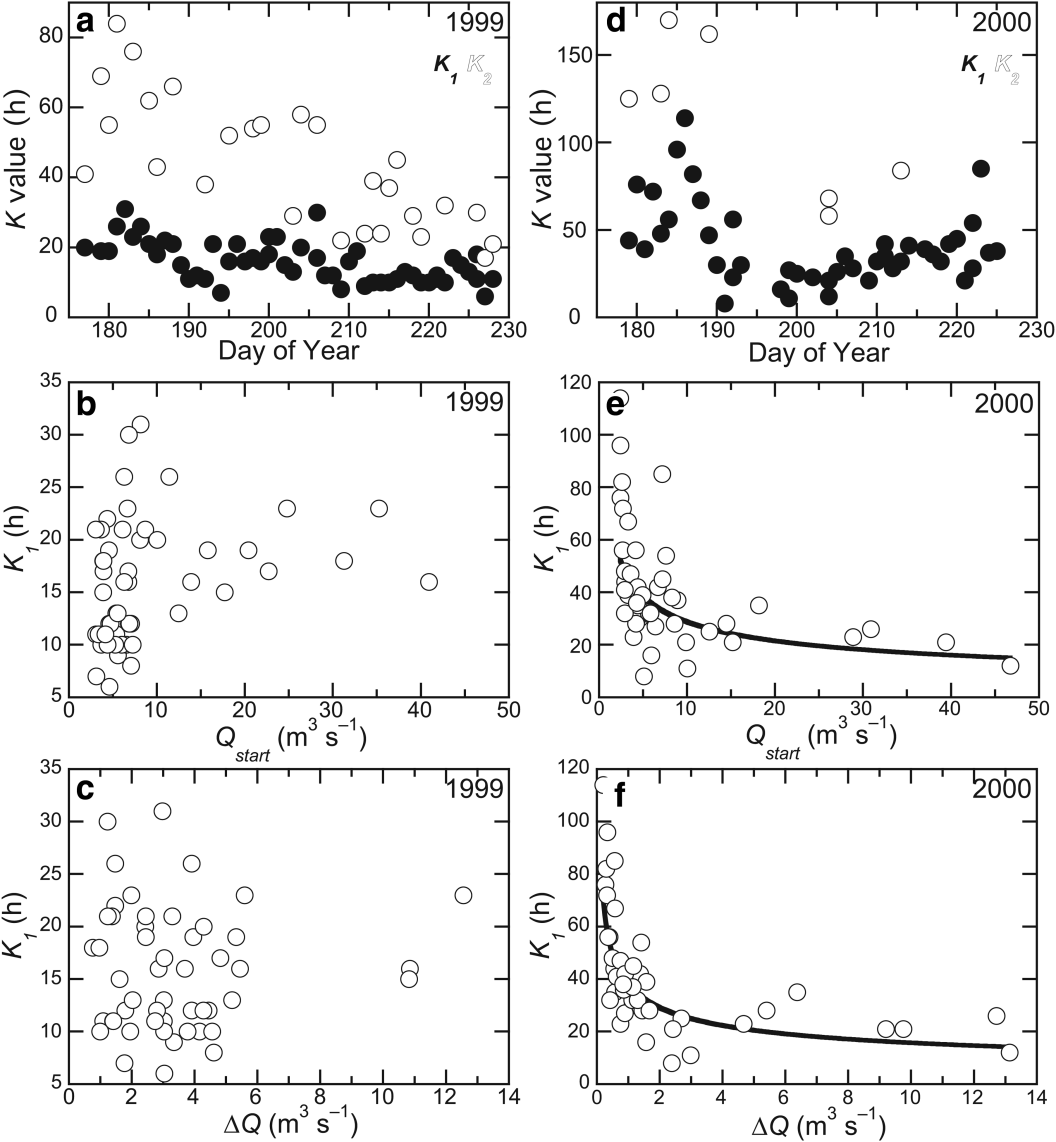
Reservoir coefficients' variation with time, start discharge (*Q*_start_) and discharge change (*ΔQ*) in 1999 and 2000. (a) The decline of coefficients with time is significant in 1999: *K*_1_ = 62–0.22*d* (*R*^2^ = 0.34), *K*_2_ = 218–0.86*d* (*R*^2^ = 0.59) where *d* is Day of Year. There is no relationship between *K*_1_ and *Q*_start_ (b) or *ΔQ* (c) in 1999. (d) The decline of coefficients with time is also significant in 2000: *K*_1_ = 146–0.52*d* (*R*^2^ = 0.10), *K*_2_ = 623–2.63*d* (*R*^2^ = 0.60). Power curves can be fitted to the relationships between *K*_1_ and start discharge (e, *K*_1_ = 76 *Q*_start_ – 0.42, *R*^2^ = 0.38), and discharge change (f, *K*_1_ = 38*ΔQ* – 0.38, *R*^2^ = 0.64).

[25] Seasonal variations in reservoir coefficients are illustrated in Figure 4. There was a net decline in the values of both *K*_1_ and *K*_2_ during both melt seasons (Figures 4a and 4b), though again there are contrasts between the two years. In 1999, the value of *K*_1_ declines at a rate of about 0.22 h d^−1^ (from about 22 to 10 h; Figure 4a); the value of *K*_2_ declines at a rate of about 0.86 h d^−1^ (from about 68 to 21 h; Figure 4a). In 2000, there is also a net decline in the value of *K*_1_ of about 0.52 h d^−1^ (from about 53 to 29 h), but this is comprised of a fairly steep decline in the first 16 days of the series and a slow increase for the remaining 30 days (Figure 4d). The transition from declining to increasing *K*_1_ does not correspond with a distinct event in the flow series, although it does occur at about the same time as flow values generally start to increase as the seasonal maximum approaches. *K*_2_ declines steeply in 2000, at a rate of about 2.6 h d^−1^ (from about 152 to 31 h; Figure 4d), but again, this does not so much reflect a steady trend as a bipartite clustering of early-season, high values and late-season, low values. Therefore, there is an overall trend in both melt seasons toward lower values of reservoir coefficients, representing faster-draining/more efficient systems, but this is not necessarily achieved in a uniform, linear fashion.

[26] No relationship is apparent in 1999 between *K*_1_ and either the flow at the start of each recession, *Q*_start_, or the total change in flow during each recession, *ΔQ* (Figures 4b and 4c). For 2000, power curves can be fitted to the *K*_1_−*Q*_start_ and *K*_1_−*ΔQ* relationships (Figures 4e and 4f). However, while there are fewer high values of *K*_1_ for high values of both *Q*_start_ and *ΔQ* (Figures 4e and 4f), there is very high scatter at low values of these flow variables, such that it is difficult to discern a satisfactory, predictive relationship. In terms of physical interpretation, it is difficult to be confident whether *K*_1_ really is highly sensitive to flow values, whether the flow-recession analysis actually captures the most appropriate values to describe the relationship between flow magnitude and throughflow rate, or whether there are shortcomings in the conceptualization of the glacier drainage system as linear reservoirs. *Gurnell* [1993] found that reservoir coefficients for different reservoirs were broadly dependent on *Q*_start_ at Haut Glacier d'Arolla (*R*^2^ = 0.11–0.48 in linear regression); it was noted that the discharge dependence of the coefficients implies that the reservoirs are not truly linear after all—although they are sufficiently linear for the purposes of hydrological simulation [*Purcell*, 2006]. However, the discharge dependence of reservoir coefficients is much less clear at Finsterwalderbreen: this may imply a drainage system which adjusts less to seasonal forcing.

### 3.3 Implied Input from Linear-Reservoir Simulation

[27] Equation (7) shows that runoff from a glacier drainage system can be simulated as one or more linear reservoirs, given a coefficient for each reservoir, an estimate of the proportion of flow routed through each reservoir, an initial value of runoff, and an input series (which mostly consists of surface melt, plus rainfall). Reservoir coefficients and flow proportions have here been determined from the flow-recession analysis; the continuous runoff series which were the subject of the analysis are of course also available. In situ meteorological or melt rate data for both years are not available; any melt modeling for this location would be highly uncertain as a result. Therefore, rather than calculate runoff, which is already known, equation (7) is here used to make a best estimate of the input series, for use in a sensitivity analysis: this is referred to as implied input, since in this instance it must consist not only of melt plus rainfall, but probably also any change in meltwater storage, particularly the release of snowmelt stored earlier in the summer [*Jansson et al*., 2003]. A lumped approach is taken here because data on the distribution of firn, snow, and ice are unavailable. However, given that the aim is specifically to evaluate the effects of reservoir characteristics, this parsimonious approach is appropriate.

[28] To calculate implied input, the observed number of reservoirs, reservoir coefficients for each recession and flow fraction to each reservoir from the flow-recession analysis (Table1) are used: as these values were, necessarily, only determined for intervals of decreasing runoff, a geometric interpolation [*Stineman*, 1980] is employed to synthesize continuous, hourly series of *K*_1_, *K*_2_, and fraction of flow in reservoir 1, *f*. Implied input is therefore still only an estimate, as the variables are partly constrained by observation, but partly interpolated. The complete equation for a two-parallel reservoir model is: 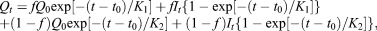
(11)

[29] The spatially averaged 1999 implied input is equivalent to 0.98 m w.e. over 54 days; the 2000 value is 0.95 m w.e. over 46 days. These values compare favorably with the 1.68 m w.e. surface melt measured over the same period at the very terminus in 1999 [*Hodgkins et al*., 2009], and with previously measured, spatially averaged summer balances of −1.15 m w.e. (1994) and −1.02 m w.e. (1995) [*Hagen et al*., 2000].

**Figure 5:**
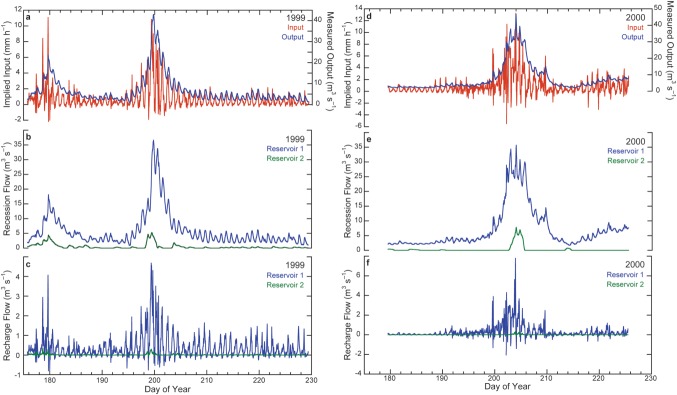
Best-fit linear-reservoir models for 1999: (a) implied input, (b) recession flow from both reservoirs, (c) recharge flow from both reservoirs. Best-fit linear-reservoir models for 2000: (d) implied input, (e) recession flow from both reservoirs, (f) recharge flow from both reservoirs. Note the changing scales between plots. Measured output is runoff (Figure 2). Implied input is that required to match measured runoff, with parameters from the flow-recession analysis, in equation (11). When the calculated recession flow is greater than the measured runoff, the implied input must be negative. This likely arises due to mis-estimation of interpolated parameters, which is probably exacerbated during the release of water stored in the glacier before monitoring began.

[30] The results of simulations using implied input (melt plus rainfall plus change in water storage, expressed as a flow rate) and values of *K*_1_, *K*_2_, and *f* calibrated from the flow-recession analysis are summarized in Figure 5 and Table2. Values of *E* show that the goodness-of-fit of the simulations is very high, which is to be expected as implied input has been determined from measured runoff; *E* would certainly be lower if the simulations were using estimated surface melt as input (either modeled, cf. *Klok et al*. [2001], or extrapolated from in situ measurements, cf. *Hannah and Gurnell* [2001]). RMSE varies from 9.8 to 22.3% of mean, seasonal runoff (Table 2). Simulations for 1999 using a single reservoir (i.e., *f* = 1.0) give only marginally poorer forecasts than those using two: this is certainly because the volume of flow in reservoir 2 is very small. For 2000, using a single reservoir actually yielded a fractional improvement compared to the two-reservoir simulation, presumably as a result of the limitations of the approximation and interpolation of *f*. The volume of flow in reservoir 2 is even smaller in 2000 than in 1999 (Table 1).

**Table 2:** Results of the Linear-Reservoir Modeling Sensitivity Analysis, Using Different Combinations of Reservoir Coefficients (*K*_1_, *K*_2_) and Reservoir Proportion of Total Flow (*f*)[Table-fn tf2-1]

Year	*K*_1_	*K*_2_	*f*	% Measured Total	ME	RMSE	*E*
1999	16	54	0.95	100.5	−0.04	0.66 (9.8)	0.99
1999	16	54	1.00	100.5	−0.04	0.73 (10.9)	0.99
2000	41	114	0.98	99.4	0.05	1.71 (22.3)	0.96
2000	41	114	1.00	99.4	−0.04	1.67 (21.7)	0.96
2000	16	54	0.95	101.0	−0.08	1.30 (16.9)	0.98
2000	16	54	1.00	101.0	−0.08	1.39 (18.1)	0.97

The figures in brackets in the RMSE column are RMSE as proportion of mean seasonal discharge.

### 3.4. Sensitivity Analysis of Linear-Reservoir Simulations

**Figure 6:**
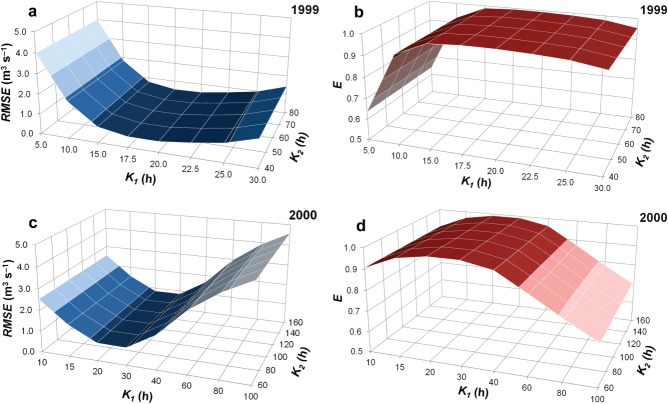
Results of the linear-reservoir modeling sensitivity analysis [after *Hock and Noetzli*, 1997]. RMSE and *E* variation for a range of combinations of *K*_1_ and *K*_2_ in 1999 (a and b, respectively), and 2000 (c and d).

[31] The implied input series (Figure 5) were then used in a sensitivity analysis, in order to evaluate how responsive the simulated runoff is to changing values of the reservoir coefficients, number of reservoirs, and reservoir flow proportions. Circularity in the sensitivity calculations is avoided as the combinations of parameters used are wholly different from those used to determine implied input. For the 1999 series, *K*_1_ varied from 5 to 30 h and *K*_2_ from 40 to 80 h, reflecting the range of values encountered from the recession analysis. For 2000, *K*_1_ values of 10−100 h and *K*_2_ values of 60−160 h were used for the same reason. The results of the sensitivity analysis are summarized in Figure 6. Simulated runoff in either year is very insensitive to the choice of *K*_2_, which is not surprising given the very low proportion of flow routed through that reservoir, even in the year (1999) when it is present on the majority of days: with *K*_1_ = 15 h, the RMSE of simulated 1999 runoff with *K*_2_ varying from 40 to 80 h only changes from 0.77 to 0.80 m^3^ s^−1^, or for *K*_1_ = 40 h, with *K*_2_ varying from 60 to 160 h, the RMSE of simulated 2000 runoff only changes from 1.62 to 1.64 m^3^ s^−1^. That is to say, for *at least* a doubling of *K*_2_, RMSE changes only by about 1−4%. *E* is similarly insensitive to *K*_2_, and varies only from 0.92 to 0.99 for *K*_1_ = 10−25 h, for all values of *K*_2_ in 1999, or from 0.96 to 0.99 for *K*_1_ = 15−40 h, for all values of *K*_2_ in 2000. The sensitivity of simulations to *K*_1_ is considered below, in relation to seasonal variability.

[32] Overall, the optimum combination of reservoir coefficients, judged from runoff simulations that yield minimum RMSE and maximum *E* when compared with the measured series, is *K*_1_ = 17−18 h, *K*_2_ = 60−80 h for 1999, and *K*_1_ = 30 h, *K*_2_ = 60−80 h for 2000 (although the 2000 runoff series was effectively as well simulated with one reservoir as with two). It therefore appears that somewhat different values of reservoir coefficients are required in order to simulate runoff successfully in consecutive years. However, the simulations have a low sensitivity to a relatively wide range of coefficient values around the optimum, so this difference is not necessarily as great as it initially appears. Seasonal and inter-annual variability in Finsterwalderbreen's drainage system are discussed further in the following sections.

## 4. Discussion

### 4.1 Drainage-System Structure

[33] The linear-reservoir model is a conceptual one, which does not ascribe specific, physical interpretations to the reservoirs themselves. It is nevertheless straightforward to assign such interpretations to fast and slow reservoirs in a general, glacial context: fast reservoirs are most likely to be characterized by supraglacial, englacial and perhaps ice-marginal routing, by efficient, channelized subglacial routing, or by some combination of these; slow reservoirs are more likely to be characterized by generally Darcian flow through snow and/or firn at the surface or through a permeable substrate, by inefficient, distributed subglacial routing, or again by some combination of these. In a spatially distributed model, the physical interpretation of the reservoir can be made explicit, by associating a particular location relative to the transient snowline with a specific coefficient. In a spatially lumped model, such as here, the reservoir effectively integrates drainage pathways from source to outflow: in the absence of spatial differentiation, and necessarily if reservoirs are arranged in parallel, each reservoir must represent a complete cascade from meltwater generation, to throughflow by one or more pathways, to runoff.

[34] There is independent evidence for different hydrological reservoirs at Finsterwalderbreen. *Wadham et al*. [2001] found meltwater solute composition during the peak discharge in 1999 (on 18 July, day 200: Figure 2) indicative of the release of snowmelt from storage; together with concurrent increased suspended-sediment concentrations [*Hodgkins et al*., 2003], this suggested that snowmelt accessed an anoxic chemical weathering environment, characterized by high-rock:water ratios and long rock-water contact times, consistent with a subglacial origin. The release was understood to be forced by an episode of rapid surface meltwater production, leading to an increase in subglacial water pressure, forcing a hydrological connection between an expanding subglacial reservoir and the ice-marginal channel system. As discharge rises rapidly to the seasonal peak, the value of *f*, representing the proportion of water routed through the fast reservoir, falls from 1.0 to 0.79 over 16−17 July, before returning to 1.0 on 19 July, when discharge has started falling. This would appear to support the notion of an episode of stored meltwater release, associated with a temporary routing switch (although a similar episode on 4−5 July lacks any obvious expression in the discharge series). The location of the subglacial reservoir is uncertain, though an overdeepened area upglacier of a bedrock ridge 6.5 km from the terminus [*Nuttall et al*., 1997; *Ødegård et al*., 1997] seems probable. This bedrock ridge is higher in elevation at the eastern margin than at the western margin, which may also explain why the majority of the glacier's meltwater drains to the western margin, and why this proportion is apparently increasing as the lower glacier retreats and thins: from 85% in 1970 to 91% in 1990, estimated from surface geometry [*Hagen et al*., 2000].

[35] The parallel arrangement of reservoirs appears to be appropriate for a polythermal glacier, such as Finsterwalderbreen, where there is independent evidence of contrasting drainage systems coexisting. *Wadham et al*. [2010] observed both the ice-marginal channel and the subglacial upwelling delivering meltwater to the proglacial area of Finsterwalderbreen: solute in the former was derived mainly from moraine pore waters, whereas the latter exhibited products of prolonged contact between meltwaters and subglacial sediments, anoxic processes driven by microbially generated CO_2_ and sulphide oxidation. The relative extent of drainage through each pathway varied from season to season, but both were typically present at the terminus: this suggests that they can justifiably be represented as parallel reservoirs. Similarly, *Vatne et al*. [1996] inferred the coexistence of fast (englacial) and slow (subglacial) meltwater drainage structures at the polythermal Hannabreen, Svalbard. This tentatively suggests that parallel reservoirs could be an appropriate approximation of the drainage systems of polythermal glaciers generally. Furthermore, the weak or absent discharge dependence of reservoir coefficients, plus the limited variation in the proportion of total flow in each reservoir, suggests a relatively stable drainage system at Finsterwalderbreen, although this study does not encompass a period of rapid, early-season snowline retreat.

[36] These considerations lead to the question of a physical interpretation of the fast and slow reservoirs at Finsterwalderbreen, apparent from the flow-recession analysis and used to simulate runoff here through the linear-reservoir model. It seems probable that the fast reservoir essentially represents the ice-marginal channel, but in a broad sense, including systems that are feeding meltwater to the channel. During the 1999 and 2000 time series, the channel system is dominated by icemelt, when the snowpack is already somewhat depleted. It then seems logical to speculate that the slow reservoir represents the subglacial upwelling, again in a broad sense. 6.0 km of travel, from the subglacial bedrock ridge to the terminus, in 54 h (*K*_2_ in 1999) or 114 h (*K*_2_ in 2000) implies a hydraulic conductivity of 0.015–0.031 m s^−1^. This is a faster rate than would be anticipated for Darcian flow through a saturated, subglacial sediment layer alone [*Hodgkins et al*., 2004; *Hubbard et al*., 1995; *Stone et al*., 1997], but slower than the near-subaerial rate expected in the ice-marginal channel (*c*. 0.10 m s^−1^ for the fast reservoir in 1999). This seems plausible for a system which is likely to be a composite of mainly englacial and subglacial pathways

### 4.2 Seasonal Variability

[37] The seasonal evolution of glacier drainage systems toward increasingly efficient states has important implications for the responsiveness of hydrological outputs, manifested in the form of the proglacial hydrograph [*Jobard and Dzikowski*, 2006; *Richards et al*., 1996; *Röthlisberger and Lang*, 1987], and for the rate of basal motion, which generally decreases as subglacial water pressures diminish with the development of faster meltwater throughflow and the release of stored water [*Fountain and Walder*, 1998]. Most studies that have employed the linear-reservoir approach have assumed constant reservoir coefficients, but have taken drainage system evolution into account by varying the proportion of the modeled glacier which is drained by fast or slow reservoirs. For example, *Hock and Noetzli* [1997] and *Klok et al*. [2001] subdivided their respective study glaciers into reservoirs based on their surface characteristics: a firn reservoir above the previous year's equilibrium line, a (variable) snow reservoir, defined as the snow-covered area outside the firn reservoir, and a (variable) ice reservoir, defined as the area of exposed ice. As the snowline retreats seasonally and more ice is exposed, more surface melt is routed to the faster-draining ice reservoir at the expense of the slower-draining snow reservoir, accounting for the seasonal evolution of the drainage system and producing more peaked diurnal hydrographs.

[38] Drainage evolution can also be inferred from flow recessions: *Hannah and Gurnell* [2001] found coefficient values for a fast reservoir declined from 13 to 5 h, and for a slow reservoir from 45 to 19 h, over a melt season at the temperate Taillon Glacier, French Pyrénées. Other studies of temperate glaciers have revealed similar coefficient decline [*Elliston*, 1973; *Collins*, 1982; *Gurnell*, 1993]. On the other hand, *Rutter et al*. [2011] found no apparent seasonal trend in reservoir coefficients at the nontemperate Rieperbreen-Foxfonna, and no significant correlation between coefficients and the sum of daily air temperatures, solar radiation or discharge at the start of each recession. *Irvine-Fynn* [2008] also found no temporal trends in reservoir coefficients at the polythermal Midtre Lovénbreen; the inferred lack of drainage development there was supported by the results of dye-tracing tests, which showed no increase in flowpath efficiency. This study has shown, more in common with the temperate examples, that the coefficient of the fast reservoir at Finsterwalderbreen declined from 22 to 10 h (1999) and from 53 to 29 h (2000), and of the slow reservoir from 68 to 21 h (1999) and from 153 to 31 h (2000), although the decline is not necessarily linear or simple. *Hock and Jansson* [2005] defend the use of constant reservoir coefficients by suggesting that the difference between the fast reservoir and the slow reservoir in most studies is sufficiently pronounced that effects from the seasonal evolution of drainage efficiency are masked. This is the case if input can realistically be apportioned between reservoirs, for example on the basis of the snowline position. However, in the spatially lumped approach taken here, input was instead apportioned according to the contributions of each reservoir to total flow, indicated by the recession analysis.

[39] While the general decline in values of reservoir coefficients in both melt seasons at Finsterwalderbreen indicates an overall increase in the efficiency of the glacier drainage system, the extent of the observed increase is limited by several factors: (1) the runoff series in both years were acquired from an interval when snow had already cleared from the lower glacier, so (presumably) large changes associated with the depletion of that meltwater source are absent; (2) the proportion of flow routed through the slow reservoir is consistently very small; (3) even though the model simulation is more sensitive to values of *K*_1_ than of *K*_2_, simulated runoff still only has a moderate sensitivity to the range of variation in *K*_1_ encountered during the main part of the melt season. Eighty percent of the 1999 melt season exhibited *K*_1_ values between 10 and 25 h, where runoff simulation RMSE only varies between 0.53 and 1.84 m^3^ s^−1^ for *K*_2_ = 50 h (Figure 6, Table 2). Likewise, 78% of the 2000 melt season showed *K*_1_ values between 15 and 50 h, where RMSE only varies between 0.84 and 2.34 m^3^ s^−1^ for *K*_2_ = 100 h (Figure 6, Table 2). Therefore, the range of variation in RMSE, associated with the variation of reservoir coefficients over the main part of the melt season, corresponds to no more than 19.6% of seasonal mean discharge, which is close to or within the range of the runoff measurement uncertainty.

### 4.3 Inter-Annual Variability

**Figure 7:**
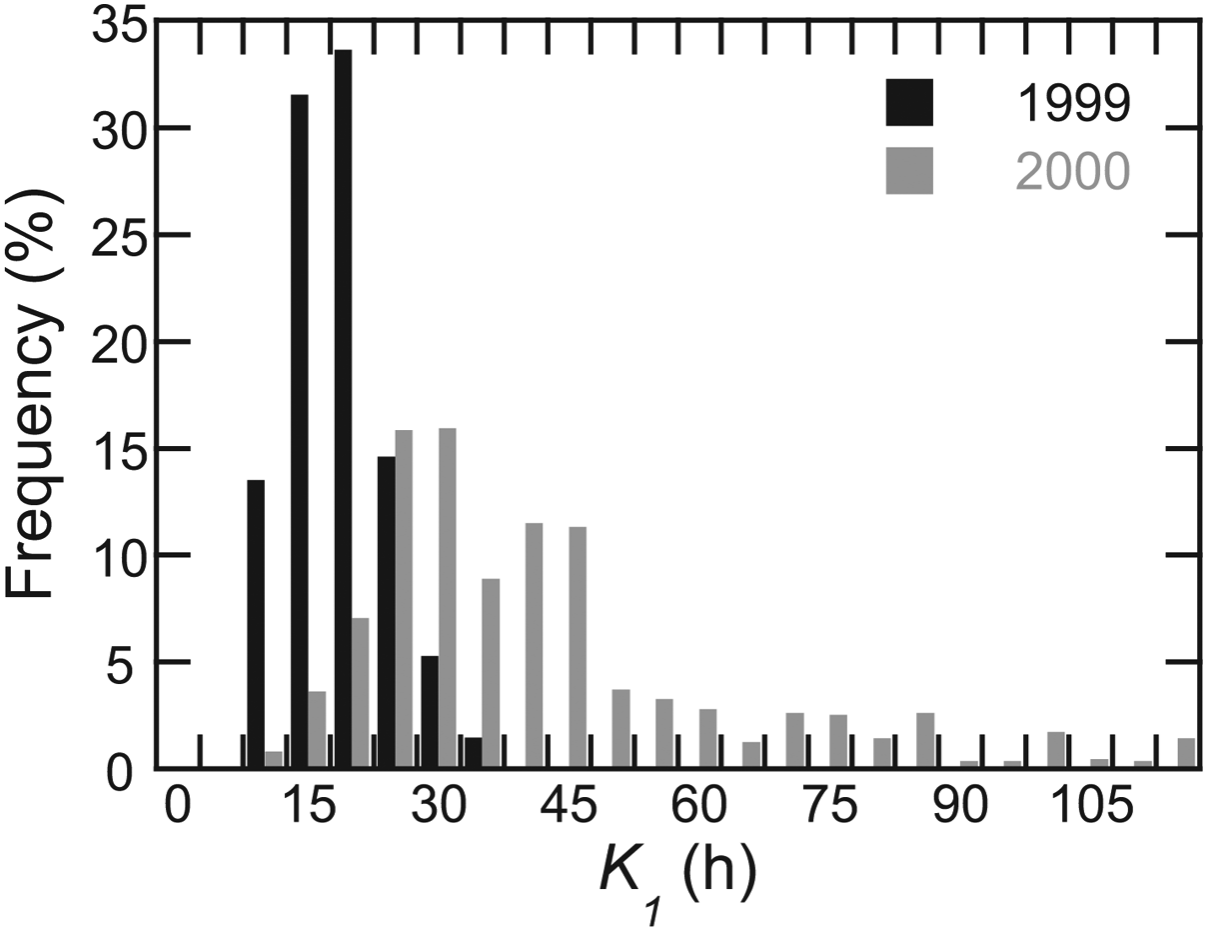
Frequency distributions of *K*_1_ in 1999 and in 2000.

[40] The difference in the prevalence of two-reservoir recessions and in the values of reservoir coefficients between the two melt seasons have been highlighted above. Likewise in Svalbard, *Irvine-Fynn* [2008] found *K*_1_ = 24 h and *K*_2_ = 33 h in one melt season at Midtre Lovénbreen, but *K*_1_ = 38 h and *K*_2_ = 86 h in the following, suggesting slower overall rates of drainage. The frequency distributions of *K*_1_ at Finsterwalderbeen in 1999 and 2000 are shown in Figure 7 (*K*_2_ distributions are not analyzed further because the slow reservoir plays such a small role in the drainage system overall). When testing the statistical significance of the difference between the *K*_1_ means in the two years, it is important to note that the 2000 frequency distribution is positively skewed (Figure 7). A *t*-test on values which are ranked, and therefore less likely to be affected by the long tail of high coefficients [*Conover and Iman*, 1981], indicates that the null hypothesis of no significant difference must be rejected at *p*<0.05 (*t* = 3.52, *t*_crit_ =, 2.02, *df* = 40, *p* = 0.001, two-tailed).

**Figure 8:**
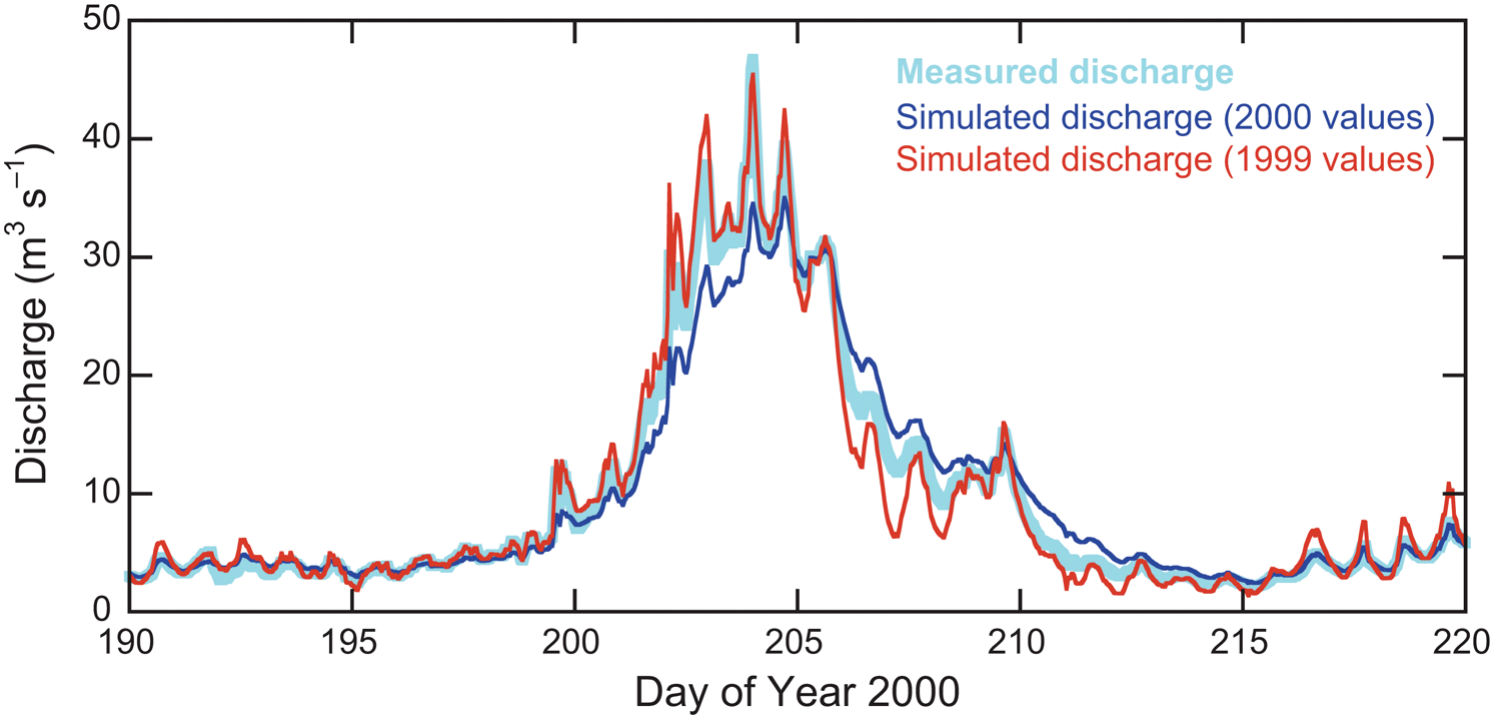
Part of the 2000 discharge time series (cf. Figure 2) simulated with linear-reservoir model values from 1999 (*K*_1_ = 16, *K*_2_ = 54, *f* = 0.95) and from 2000 (*K*_1_ = 41, *K*_2_ = 114, *f* = 0.98).

[41] The statistically significant difference in the means of the reservoir coefficients in consecutive years suggests appreciable inter-annual variability in drainage-system efficiency (represented by the rate of meltwater throughflow), if not in drainage-system structure. However, the limited sensitivity of simulated runoff to a wide range of reservoir coefficients limits the significance of this result for simulation purposes: simulation of 2000 runoff using mean, flow-recession-calibrated parameters from 1999, gives results that are not appreciably worse than using the equivalent 2000 parameters (Table 2). This very similar overall performance tentatively suggests that reservoir coefficients can after all be transferred between melt seasons. The simulated 2000 runoff series using 1999 parameters does not, unsurprisingly, represent the diurnal cycling of the early and late season as well as a simulation using mean parameters from 2000 itself, but it does, more surprisingly, better represent seasonal peak discharge and its decline over the interval 20 July–1 August (days 202−214: Figure 8). The explanation for both the diurnal-cycle overprediction, and the successful capture of seasonal peak discharge and decline, is that the 1999 *K*_1_ (16 h) generates faster throughflow than its 2000 equivalent (41 h), enhancing the amplitude of regular diurnal cycles but effectively capturing the secular trend in late July, an interval in which 2000's own *K*_1_ values indicated by the flow-recession analysis are steadily increasing from about 20 to about 40 h (Figure 4d). Rather than representing a fundamental shortcoming with the flow-recession analysis or with the linear-reservoir approach, this case highlights the limitation of using a constant coefficient for the fast reservoir in a spatially lumped simulation. The key determinant of coefficient applicability will be the timescale of interest (subseasonal, seasonal, multiseasonal).

[42] The greater mean values of reservoir coefficients indicate that the glacier drainage system is draining more slowly on the whole in 2000 than in 1999. This is a likely explanation of why many fewer slower-reservoir components are seen in the flow recessions from 2000 (Table 1): the lower rate of drainage in the faster reservoir means that few recessions are completed before the next rise in the hydrograph. It also makes better physical sense for the slow-reservoir outflow to be obscured by fast-reservoir outflow, rather than for the actual slow component itself to disappear and reappear. From a flow-recession analysis of the 1989 melt season at Haut Glacier d'Arolla, *Gurnell* [1993] noted that diurnal cycles appeared on 11 June, but that breaks of slope in recessions, indicating the presence of a fast reservoir, did not appear until 2 July. By the time monitoring started in both years at Finsterwalderbreen, diurnal cycles were established in the runoff series, though subdued compared to their later occurrence, and the fast reservoir was already present.

[43] The simplest explanation for the lower values of the reservoir coefficients in 2000 is that the snow cover was more persistent in that melt season. As snowmelt is feeding both faster and slower reservoirs, both coefficients are greater, although it is expected that snowmelt is proportionally more significant in the slower reservoir than the faster one. Snow extent time series are not available for these melt seasons, but there are data to support this interpretation: *Hodgkins et al*. [2005] found that the mean spring snow depth on Finsterwalderbreen in 2000 (0.58 m w.e.) was greater than in 1999 (0.41 m w.e.), while *Luks et al*. [2011] observed that the 2000 melt season started later than the 1999 one at Hornsund, less than 50 km south of Finsterwalderbreen.

## 5. Conclusions

[44] The hydrological significance of glaciers, and the responsiveness of ice flow to the mode of glacier meltwater drainage, indicates that that more detailed understanding of the drainage systems of glaciers is required. This is particularly true of high-latitude glaciers with polythermal temperature regimes, since these are not only less well-studied than their temperate counterparts, but are more likely to influence the stability of the ice sheets [*Lemke et al*., 2007]. Flow-recession analysis and linear-reservoir simulation of runoff time series from consecutive years at Finsterwalderbreen have yielded the following insights into the seasonal and inter-annual variability of that glacier's drainage system.

[45] Linear flow recessions are pervasive features of the runoff series. Two-reservoir recessions, consisting of a faster component followed by a slower component, characterize 1999, whereas one-reservoir recessions are typical of 2000. The coefficients of the faster reservoir differ significantly between the years: 16 h in 1999, 41 h in 2000. This is a probable explanation of why many fewer slower-reservoir components are seen in the flow recessions from 2000: the faster components which occur at the start of each recession take longer to complete. There is an overall trend in both melt seasons toward decreasing values of reservoir coefficients (0.22 h d^−1^ in 1999 and 0.52 h d^−1^ in 2000), indicating that drainage efficiency increases seasonally, though this is not necessarily achieved progressively, and while the reservoir coefficients generally decline, they do not exhibit a consistent relationship with discharge. In this respect, Finsterwalderbreen behaves as an intermediate case between temperate glaciers and other polythermal (with smaller proportions of temperate ice) and nontemperate glaciers, that have been similarly studied in Svalbard to date.

[46] The consistent identification of reservoirs from flow-recession analysis means that runoff can be successfully simulated with the linear-reservoir approach. Results obtained using only a single reservoir are almost as good as those obtained with two, because of the very low volume of flow that actually occurs in the slow reservoir each year (no more than 5% on average in 1999, or 2% in 2000). Simulations also have a low sensitivity to a relatively wide range of coefficient values around the optimum: the range of variation in RMSE of simulated runoff, associated with the variation of reservoir coefficients, is comparable to the runoff measurement uncertainty in this challenging environment for hydrometry. Nevertheless, the use of constant reservoir coefficients in a spatially lumped model can diminish the performance of the simulation at subseasonal timescales.

[47] The greater mean values of reservoir coefficients in 2000 indicate that the glacier drainage system is on the whole draining more slowly than in 1999. There is no indication that the drainage-system structure is essentially different between the two years: the simplest explanation for the lower efficiency in 2000 is that the snow cover was more persistent in that melt season, so that slow percolation through snow forms a greater proportion of overall flow pathways. The parallel arrangement of reservoirs in a linear-reservoir model appears to be appropriate for a polythermal glacier, where there is evidence of contrasting flow pathways concurrently delivering meltwater to the glacier terminus. In the case of Finsterwalderbreen, it appears that the fast reservoir generally corresponds to an ice-marginal channel, and the slow reservoir to a subglacial upwelling. By routing icemelt to the glacier margin, and snowmelt subglacially, nontemperate ice appears to allow flow pathways of very different efficiencies (and therefore, presumably, water pressures) to exist in relatively close proximity throughout the melt season: a significant difference from temperate systems.
